# Deciphering the regulatory role of *PheSnRK* genes in Moso bamboo: insights into hormonal, energy, and stress responses

**DOI:** 10.1186/s12864-024-10176-7

**Published:** 2024-03-06

**Authors:** Huifang Zheng, Yali Xie, Changhong Mu, Wenlong Cheng, Yucong Bai, Jian Gao

**Affiliations:** 1 Key Laboratory of National Forestry and Grassland Administration/Beijing for Bamboo & Rattan Science and Technology, International Center for Bamboo and Rattan, State Forestry and Grassland Administration, 100102 Beijing, China; 2 College of Life Science, Leshan Normal University, Leshan, China

**Keywords:** Low energy, Protein kinase, Salt stress, Sucrose metabolism

## Abstract

The SnRK (sucrose non-fermentation-related protein kinase) plays an important role in regulating various signals in plants. However, as an important bamboo shoot and wood species, the response mechanism of *PheSnRK* in *Phyllostachys edulis* to hormones, low energy and stress remains unclear. In this paper, we focused on the structure, expression, and response of SnRK to hormones and sugars. In this study, we identified 75 *PheSnRK* genes from the Moso bamboo genome, which can be divided into three groups according to the evolutionary relationship. *Cis*-element analysis has shown that the *PheSnRK* gene can respond to various hormones, light, and stress. The PheSnRK2.9 proteins were localized in the nucleus and cytoplasm. Transgenic experiments showed that overexpression of *PheSnRK2.9* inhibited root development, the plants were salt-tolerant and exhibited slowed starch consumption in *Arabidopsis* in the dark. The results of yeast one-hybrid and dual luciferase assay showed that PheIAAs and PheNACs can regulate *PheSnRK2.9* gene expression by binding to the promoter of *PheSnRK2.9*. This study provided a comprehensive understanding of *PheSnRK* genes of Moso bamboo, which provides valuable information for further research on energy regulation mechanism and stress response during the growth and development of Moso bamboo.

**Supplementary Information:**

The online version contains supplementary material available at 10.1186/s12864-024-10176-7.

## Introduction

China is rich in bamboo resources, bamboo species account for about one half of the world’s total, covering approximately one-third of the global area [1]. According to statistics, by the end of June 2022, there were 251 cultivated varieties of 47 genera and 770 species of bamboo in China [1]. Bamboo is one of the plants with high carbon sequestration potential [2, 3]. The carbon sequestration capacity of bamboo forest surpasses that of ordinary trees. The annual carbon sequestration capacity of bamboo per hectare is 5.09 tons, 1.46 times that of Chinese fir and 1.33 times that of tropical rainforest [4]. Bamboo exhibits fast growth, developed underground system and excellent wood quality. The growth height of bamboo can exceed 1 m in one day at the earliest, and it can be completed in 40 to 60 days [5]. Considerable biomass accumulation in a short period of time inevitably requires a large amount of energy supply. However, there are limited research on energy perception and regulation of bamboo. Currently, the genetic transformation system of Moso bamboo is not mature. Although there have been literature reports on the genetic transformation of Moso bamboo [6], there are still some problems such as low efficiency and long period of transformation.

Sucrose non-fermentation-related protein kinase (SnRK) plays a crucial role in regulating carbohydrate metabolism balance and abiotic stress response in plants [7]. Plant SnRK is usually divided into three subfamilies: SnRK1, SnRK2, and SnRK3, according to sequence similarity and gene structure [8]. Plant SnRK1 shares strong homology with yeast’s SNF1 and the animal AMP-activated protein kinase [9]. As an evolutionarily conserved energy-sensing kinase, plant SnRK1 protein kinase coordinates transcriptional regulatory networks to maintain cellular energy homeostasis during energy scarcity, serving as a pivotal factor in plant energy deficiency perception [10]. In plants, SnRK1 not only modulates energy responses under adverse conditions but also contributes to nutrient allocation between source reservoirs and additional functions [11]. Compared with the SnRK1 subfamilies, the SnRK2 and SnRK3 subfamilies, which are unique to plants, display greater variability in gene structure and function. They are involved in the regulating a broad spectrum of biological processes. The SnRK2 subfamily features a conserved P-kinase domain (P) along with a C-terminal variable regulatory domain [12], whereas the SnRK3 subfamily possesses an N-terminal conserved protein kinase domain, an NAF domain, and a C-terminal PPI domain [13].

A growing body of research underscores the pivotal regulatory function of the SnRK1 protein kinase in plants’ perception of energy scarcity [14, 15]. Sucrose instigates the swift dephosphorylation of SnRK1 targets [16]. In *Arabidopsis* rosette leaves, SnRK1 modifies the interplay between sucrose and trehalose 6-phosphate (Tre6P), impacting the transformation of sucrose into Tre6P accumulation and directing carbon flow within the TCA cycle downstream of the Tre6P signaling pathway [17]. Under optimal culturing conditions devoid of external stimuli, SnRK1 is instrumental in maintaining sucrose homeostasis and remodeling the transcriptome in rosettes, its activity being modulated by the diurnal variations in Tre6P levels [17].

Furthermore, the SnRK2 family is integral to abscisic acid (ABA) signaling and the response to environmental stressors. In *Arabidopsis thaliana*, the isoforms *SnRK2.2*, *SnRK2.3*, and *SnRK2.6* are markedly upregulated by ABA, central to the positive regulation of ABA-dependent signaling pathways [18]. SnRK2.4 and SnRK2.10, also in *Arabidopsis thaliana*, are ABA-non-responsive kinases that play a role in the regulation of reactive oxygen species (ROS) homeostasis under saline conditions [19]. The wheat *TaSnRK2.11* reacts to elevated temperatures, salinity and drought stresses [20]. In addition, SnRK2 proteins can bind to bZIP transcription factor to modulate gene expression [21, 22]. The SnRK3 subfamily, able to interact with calcineurin B-like (CBL) proteins, is referred to as CBL-interacting protein kinases (CIPKs). These CIPK proteins are well-recognized for their contributions to plant developmental, signal transduction, and abiotic stress response [23]. For instance, in *Arabidopsis thaliana*, the CBL5-CIPK11 complex phosphorylates and regulates the guard cell anion channel SLAC1, thereby playing a defensive role in the cell [24].

The SnRK2s family not only associates with ABA hormone signaling, but also interacts with other hormone signals, including IAA and GA. Specifically, the bZIP11 transcription factor, positively regulated by SnRK1, enhances the expression of INDOLE-3-ACETIC ACID INDUCIBLE 3/ SHORT HYPOCOTYL 2 (IAA3/SHY2). IAA3/SHY2 acts as a crucial inhibitor of auxin signaling, thereby restricting root growth in response to nutrient scarcity [25]. Furthermore, members of the SnRK3/CIPK family significantly influence auxin transport and signaling, which are vital for controlling root growth and development [26]. In rice, GA signaling inactivates SnRK2 family kinases through its interaction with the receptor GA INSENSITIVE DWARF 1 (GID1) [27]. Similarly, SnRK1 plays a contributory role in orchestrating JA- and SA-mediated defense mechanisms in rice [28]. The SnRK2 family also impinges upon cytokinin (CK) signaling by stabilizing the A-type response regulator 5 (ARR5) through phosphorylation [29]. Moso bamboo serves as a substantial biomass resource with considerable carbon sequestration capability [5, 30]. Owing to its rapid growth, high-yield, and superior timber characteristics, it is emerging as a significant alternative to traditional wood sources. Nevertheless, the knowledge regarding the SnRK family members in Moso bamboo remains scarce.

Hence, investigating the *PheSnRK* gene family will deepen our comprehension of the growth, development, and stress adaptation mechanisms in Moso bamboo, providing a theoretical framework and technical guidance for its breeding and production. Moreover, this research could broaden the understanding of *SnRK* gene family at large and offer insights for further exploration of the *SnRK* gene family’s role in plant growth, development, and stress adaptation.

## Materials and methods

### Database searches for SnRK genes in Moso bamboo and analyses of physicochemical characteristics

For the precise identification of the SnRK genes in Moso bamboo, rigorous searches across multiple databases were conducted following the methodology outlined by Xie [31]. Initially, mRNA sequences of SnRK from *Oryza sativa* and *Arabidopsis thaliana* were retrieved from NCBI Nucleotide database (https://www.ncbi.nlm.nih.gov/, accessed on 16 August 2022). These sequences were then utilized as query inputs to perform BLSAT searches against the Moso bamboo genome database. During the BLAST filtration, SnRK genes were pinpointed by employing SnRK mRNA sequences from additional species. A relatively liberal e-value threshold of < 0.00001 was set for this purpose. Following this, the protein sequences of the tentatively identified genes were subjected to another round of BLAST against the NCBI non-redundant protein database, this time applying a more stringent e-value of < 0.0000000001 to ensure specificity. Only those sequences characterized as SnRK proteins, or as part of the SnRK family, were retained for further analysis, while those annotated as belonging to other protein families were excluded.

After curating the sequences, the genomic sequences of Moso bamboo SnRK genes, including coding sequences, protein sequences, as well as newly predicted or previously mis-annotated SnRK genes were compiled using the Moso bamboo genome database. To gain insights into the physical properties of these proteins, molecular weights and theoretical isoelectric point (pI) were calculated via tools available on ExPASY (http://web.expasy.org/compute_pi/, accessed on 22 August 2022). Lastly, cell localization predictions for the candidate PheSnRK proteins were made using CELLO v2.5 Server (http://cello.life.nctu.edu.tw/, accessed on 22 August 2022), providing crucial information on their potential functional sites within the plant cells.

### Phylogenetic analysis

The SnRK protein sequences from *Arabidopsis thaliana* and *Oryza sativa* were used to classify and predict the functional roles of the SnRK genes in Moso bamboo. We performed multiple sequence alignments of the full-length SnRK proteins from *Arabidopsis*, rice, and Moso bamboo using ClustalX 1.83 (http://www.clustal.org/) and two online programs, Clustal Omega and MUSCLE. We constructed a neighbor-joining (NJ) phylogenetic tree with MEGA7.0, employing 1000 bootstrap replicates to validate tree topology.

### Gene structural and protein motif analyses of PheSnRK genes

To analyze exon–intron organizations and intron types, we utilized the encoding and genomic sequences of PheSnRKs with the Gene Structure Display Server (GSDS) (http://gsds.cbi.pku.edu.cn/index.php, accessed on 20 October 2022). We identified the conserved motifs within the PheSnRK sequences using MEME version 4.12.0 (http://meme-suite.org/tools/meme, accessed on 20 October 2022).

### Chromosomal location and gene duplication of *PheSnRKs*

The chromosomal positions of *PheSnRKs* genes across the 24 chromosomes of bamboo were plotted using TBtools software [32]. The multicollinearity Scan Kit (MCScanX) software facilitated our analysis of SnRK gene collinearity.

### Cis-element analysis of *PheSnRK* genes

A 2000-bp region upstream of the initiation codon of each *PheSnRK* gene was extracted from the Moso bamboo genome database. We utilized this sequences data to identify cis-acting regulatory elements using the PlantCARE online tool (http://bioinformatics.psb.ugent.be/webtools/plantcare/html/, accessed on 22 August 2022) [33].

### Expression pattern analysis

Transcriptome datasets from different developmental stages [34] and various floral organs [35] were obtained from prior investigations conducted by our research group. Bamboo shoots were selected 50 cm tall in the bamboo forest for basal injections of exogenous sugars and hormones every three days and sampled after fifteen days for transcriptome sequencing. Auxin related bamboo shoot transcriptome data are stored in the SAR database under access number PRJNA788576 [36]. Heat maps presented in this study were generated using TBtools software.

The expression patterns of *PheSnRKs* were detected in different organs of two-month-old bamboo seedlings. Quantitative real-time PCR (qRT-PCR) was employed to assess the relative expression levels of *PheSnRK1s* with *TIP41* (tonoplast intrinsic protein 41 gene) [37] serving as the internal control. Total RNA extraction was performed using the TRIzol method (Invitrogen, Carlsbad, CA, USA), and the first-strand cDNA synthesis was conducted with a First-Strand Synthesis Master Mix (LABLEAD, Beijing, China). qRT-PCR assays were performed using a LightCycler 480 Real-Time System (Roche, Rotreuz, Switzerland). The operation procedure and analysis for qRT-PCR followed themethodology outlined by Zheng [38].

### Subcellular localization of PheSnRK2.9 and heterologous transformation of *Arabidopsis thaliana*

PheSnRK2.9 was cloned into the p2300-GFP vector using M5 ligase and then transformed into *Agrobacterium tumefaciens* strain GV1301. *Agrobacterium* cultures preconditioned with an induction medium containing 10 mM MgCl_2_, 10 mM MES, and 100 µM acetogenone at pH 5.6 (OD600 = 0.8) were infiltrated into the leaves of *Nicotiana benthamiana*. Following a 48 h incubation under low-light conditions, the fluorescent signals were visualized using a confocal laser scanning microscope. Prediction of the PheSnRK2.9 protein 3D structure was conducted at http://www.sbg.bio.ic.ac.uk/phyre2/html/page.cgi?id=index.

For *Arabidopsis thaliana* transformation, Agrobacterium harboring the target gene-plasmid was deployed using the floral dip method to produce transgenic overexpression lines in Col-0 ecotype [39]. The wild-type *Arabidopsis thaliana* used in this experiment was obtained from our laboratory, and the transgenic work and identification using the wild-type background were carried out by the author of this article, Huifang Zheng. Primers sequences are provided in Supplementary Table S1, and positive transformant identification is depicted in Supplementary Figure S1.

### Phloroglucinol staining

Stem segments proximal to the base of rosette leaves, obtained from both wild-type and transgenic lines, were hand-sectioned. Sections were placed on glass slides and sequentially treated with 200 µL of acidified solution, followed rapidly by an equal volume of phloroglucinol reagent. Color development was observed, and images were captured promptly using an optical microscope. The quantification of lignin staining was executed utilizing ImageJ software [40].

### Transgenic *Arabidopsis* seeds treated with salt stress

Seeds from both wild-type and transgenic *Arabidopsis thaliana* were spot-planted on growth medium containing 0,150 mM NaCl to monitor seeding development over a 14-day period. About 35 *Arabidopsis* seeds per trial were planted on the medium containing 100 mM NaCl, and the seed germination number was counted at the same time every day, with radical breakthrough of seed coat as the criterion for germination, and the statistics were conducted for 7 consecutive days. The experiment was repeated three times.

### Statistical analysis of data

Data presented in this study represent the mean ± SE from a minimum of at least three replicates. The significance of the differences between means was assessed using one-way analysis of variance (ANOVA) complemented by the minimum significance difference (LSD) tests, considering a *p*-value of *p* < 0.05 as statistically significant. Data visualization was conducted using Origin software (OriginPro 2021b), while image composition for publication was prepared using Adobe Illustrator (AI) software. TBtools software was the platform of choice for heat map production.

## Results

### Identification and phylogenetic tree analysis of SnRK family genes in Moso bamboo

In our comprehensive search of the Bamboo Genome Database, we identified a total of 75 putative SnRK genes through local BLAST and subsequent gene annotation. Detailed sequence features and predicted subcellular localizations are listed in Supplementary Table S2. The coding sequences (CDS) of these SnRK genes ranged from 852 to 2283 bp, and amino acid residues ranged from 283 to 760aa, with relative molecular masses between 32.8 and 83.2 kDa.

**Fig. 1:**
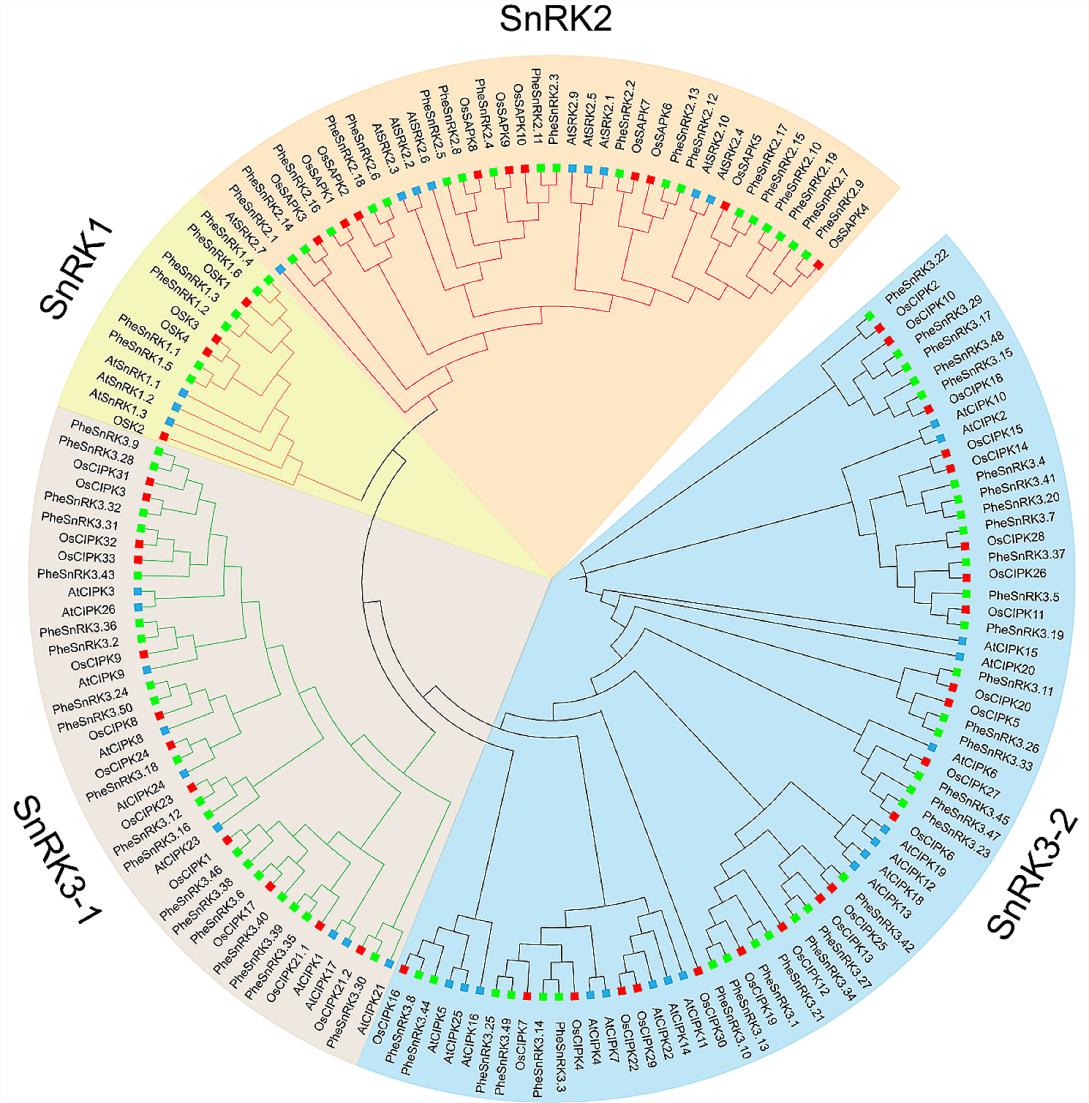
Phylogenetic analysis of SnRK proteins in *Oryza sativa*, *Arabidopsis thaliana* and *Phyllostachys edulis*. The red squares represent the SnRK family proteins of *Oryza sativa*, the blue squares represent the SnRK family proteins of *Arabidopsis thaliana*, and the bright green squares represent the SnRK family proteins of *Phyllostachys edulis*. Different subfamily branches are distinguished by adding different background colors

For insights into the evolutionary relationships of SnRK family genes, protein sequences from *Phyllostachys edulis*, *Arabidopsis thaliana*, and *Oryza sativa* were used to construct a rootless phylogenetic tree (Fig. 1). The phylogenetic analysis allowed us to categorize Moso bamboo’s SnRKs into three distinct subfamilies. The *SnRK1* subfamily comprises 6 members (*PheSnRK1.1*-*PheSnRK1.6*), each containing the Pkinase (PF00069), UBA (PF00627) and KA1 (PF02149) domains. The *SnRK2* subfamily includes 19 members (*PheSnRK2.1*-*PheSnRK2.19*), all harboring the pkinase domain. The most expansive *SnRK3* subfamily consists of 50 members (*PheSnRK3.1*-*PheSnRK3.50*), each of which features both Pkinase and NAF (PF03822) domains.

### Chromosomal distribution and synteny analysis of *SnRK* family genes in Moso bamboo

**Fig. 2:**
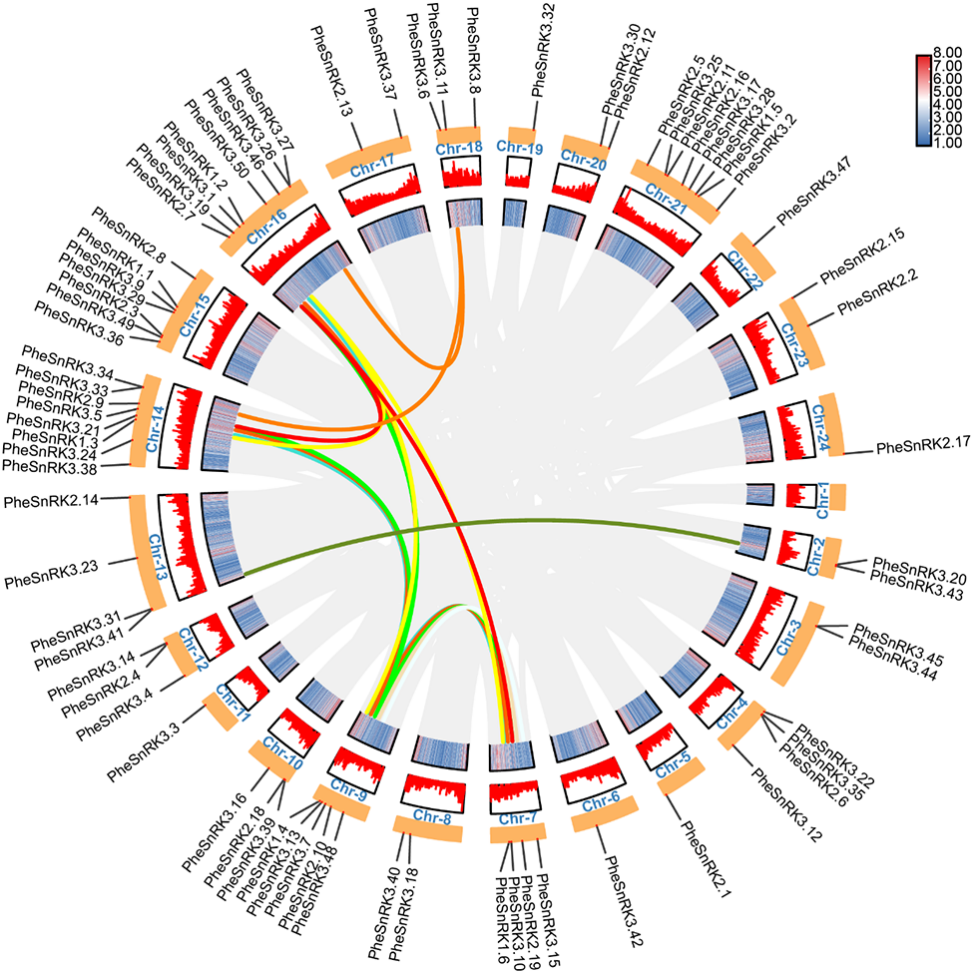
Synteny analysis of *PheSnRK* gene in *Phyllostachys edulis* genome. Gray line: All homologous blocks in the genome of *Phyllostachys edulis*. Red line: Duplicate *PheSnRK* gene pairs. The color bar represents gene density

Chromosome mapping of the 75 identified *SnRKs* genes revealed they are spread across all 23 chromosomes of *Phyllostachys edulis*, except for chromosome 1 (Fig. 2). Notably, chromosomes 1, 14 and 21 each harbored 8 *SnRK* genes, suggesting a non-specific or random distribution pattern across the chromosomes. Synteny analysis of the *Phyllostachys edulis* genome uncovered 6 pairs of *PheSnRKs* that are likely the result of gene duplicate events.

**Fig. 3:**
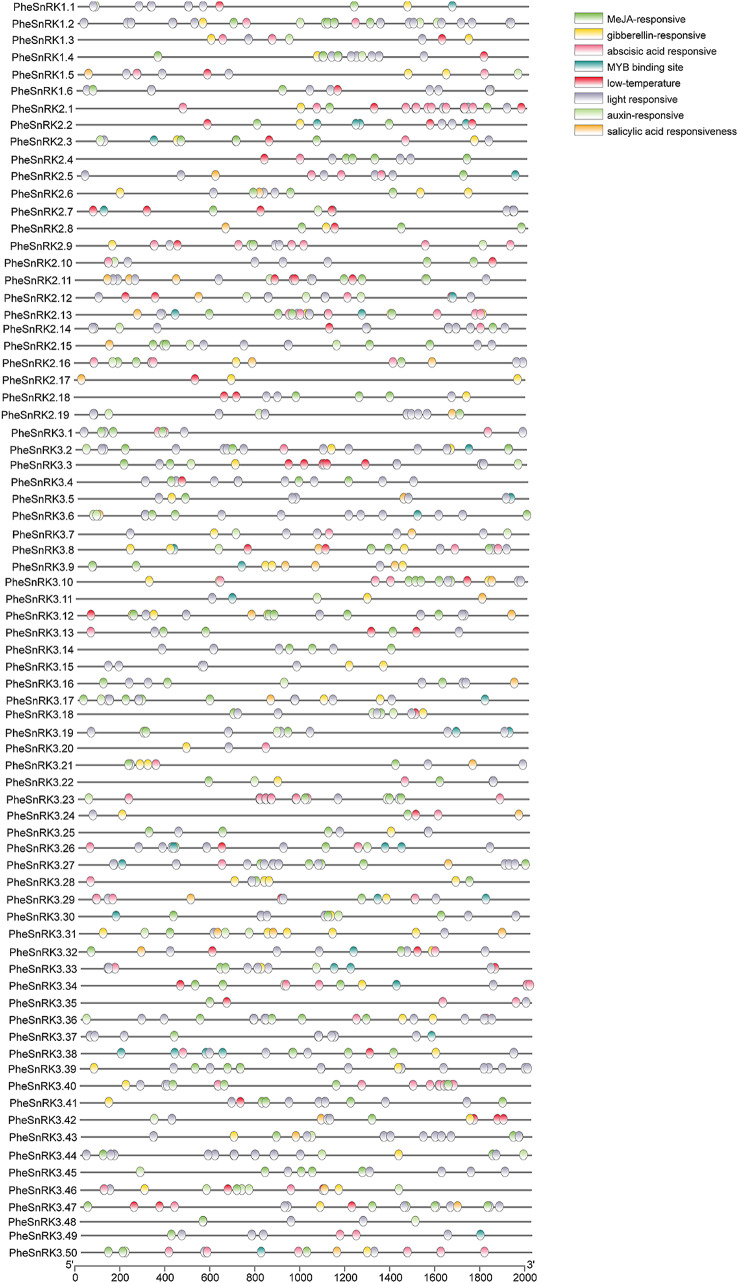
Cis-regulatory elements analysis of *PheSnRK* genes

Further, to elucidate potential regulatory mechanisms and functions of the *PheSnRK* genes, *cis*-element analysis was performed on 2000 bp sequence upstream of the start codon (ATG) using the PlantCARE database (Fig. 3; Suppl. Table S3). The identified cis-acting elements were predominantly associated with hormone responses, including auxin, ABA, gibberellic acid (GA), methyl jasmonate (MeJA), salicylic acid (SA), as well as response to low temperature and light. Remarkably, promoters of 72 out of the 75 *PheSnRK* genes featured elements responsive to light, with the promoters of *PheSnRK2.1*, *PheSnRK3.23*, and *PheSnRK3.44* each containing as many as 17 light-responsive elements. Additionally, 20 promoters encompassed 10 or more of these elements. A majority of the genes, 67 in total, exhibited promoters with elements responsive to both ABA and MeJA. This analysis suggests that the *PheSnRK* genes have the potential to respond to a multitude of hormonal signals, indicating diverse regulation across different *PheSnRK* genes members.

### Gene structure and conserved motifs analysis of SnRK family members

**Fig. 4:**
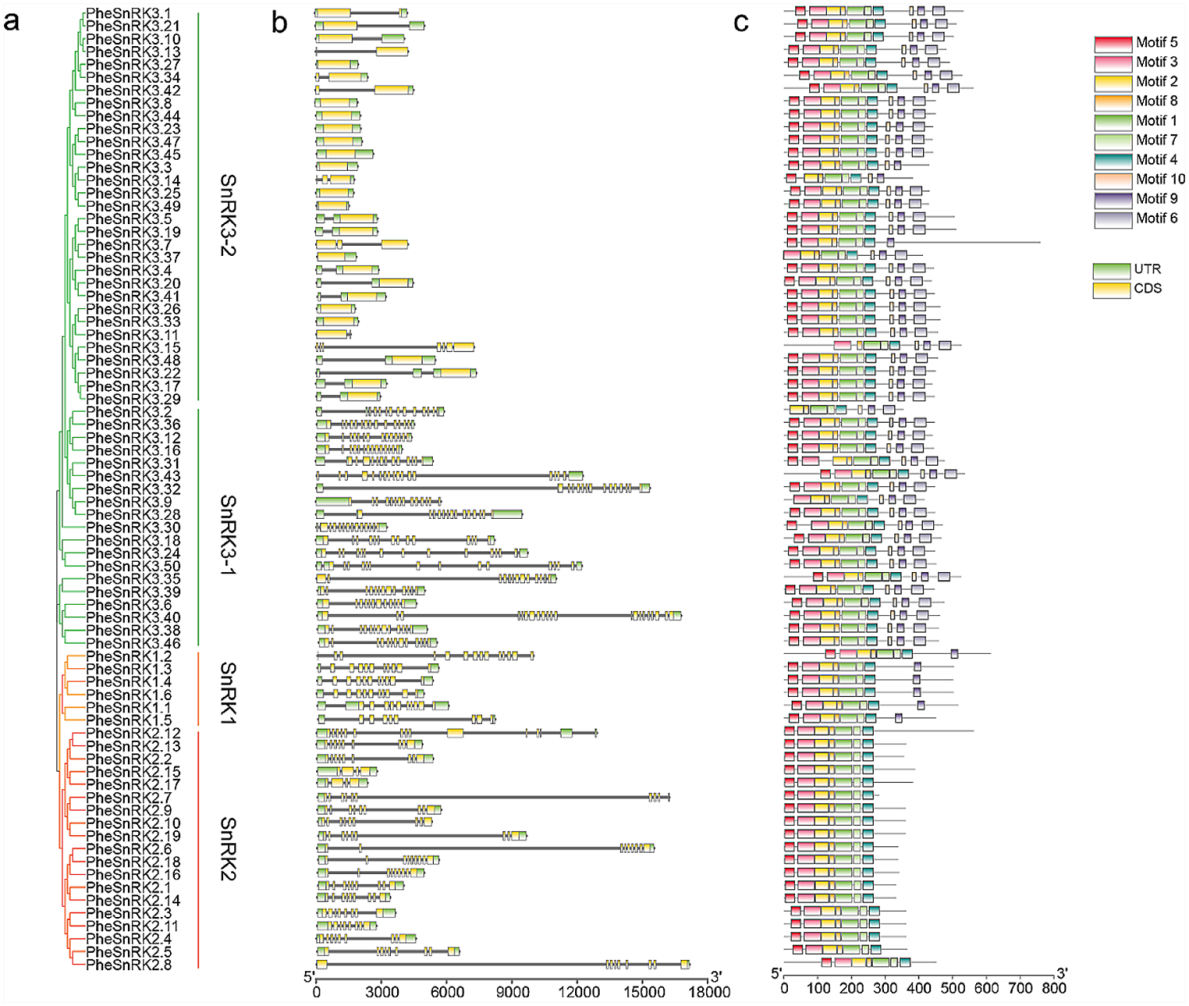
Phylogenetic relationships, gene structures and conserved motifs of the SnRK proteins in *Phyllostachys edulis*. **a** Phylogenetic tree of 75 PheSnRK proteins; **b** Gene structures of *PheSnRK* genes. Yellow boxes: exons. Black lines: introns. Green boxes: UTR areas; **c** The motif analysis of PheSnRK proteins

The gene structures of SnRK family genes were characterized using the Gene Structure Display Server (Fig. 4b). We associated the conserved domains of the proteins with their phylogenetic relationships (Fig. 4a). Our analysis revealed that the number of exons varied within the family, with 8, 10, and 13 exons identified in *PheSnRK1* gene structures; 4 to 14 exons in *PheSnRK2* gene structures; and the most considerable variation, 1to 21 exons, in *PheSnRK3* genes.

Motif composition was analyzed across all SnRK protein structures (Fig. 4c). The motifs commonly present in SnRK proteins included patterns1, 2, 3, 4, 5, 7,8. Within each subfamily, similar arrangements of conserved motifs were found. The PheSnRK1 subfamily contained motifs 1–5, 7–8, and 9; The PheSnRK2 subfamily included motifs 1–5, 7, and 8; The PheSnRK3 subfamily featured motifs 1–9. This motif distribution suggested notable conservation of gene structure within subfamilies and corroborated the phylogenetic subfamily classification’s robustness.

### Gene expression pattern analysis

**Fig. 5:**
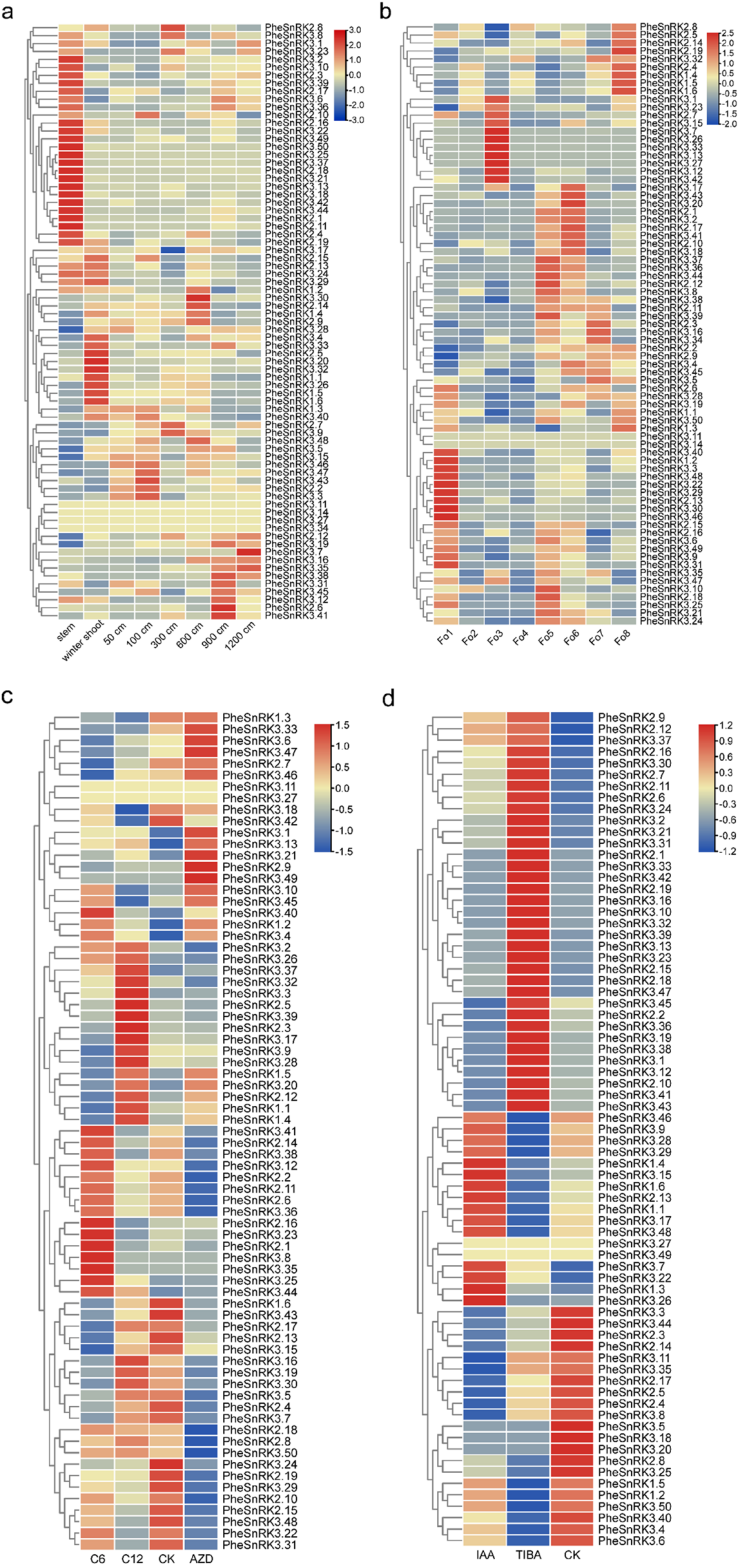
Expression profiles of *PheSnRK* genes. **a** Expression patterns of *PheSnRK* in bamboo shoots at different developmental stages. **b** Expression patterns of *PheSnRK* in different floral organs. **c** Transcriptional profiles of the *PheSnRK* genes under exogenous sugars and AZD8055. **d** Transcriptional profiles of the *PheSnRK* genes under IAA and TIBA treatments. Stem: Culms after leaf expansion; 50, 100, 300, 600, 900 and 1200 cm: The tips of bamboo shoots of different heights; Fo1: leaf; Fo2: pistil; Fo3: stamen; Fo4: young embryo; Fo5: glume; Fo6: lemma; Fo7: Flower bud; Fo8: bract; C6: glucose; C12: sucrose; AZD: AZD8055; CK: Control treatment; IAA: auxin; TIBA: triiodobenzoic acid. The color scale as in Log2 fold change relative to control treatment

Notable changes in the expression of *PheSnRK* genes were observed across various developmental stages of bamboo shoots (Fig. 5a). The expression of *PheSnRK1* family genes primarily decreased during shoot development, aside from *PheSnRK1.2* and *PheSnRK1.4*, which displayed higher expression in the shoot tips of 600 cm bamboo shoot. Selected genes from the *PheSnRK2* and *PheSnRK3* subfamilies were highly expressed in the stem. In different floral organs, most *PheSnRK* genes exhibited low expression in pistils and young embryos (Fig. 5b). Genes *PheSnRK1.1*, *PheSnRK1.2*, and *PheSnRK1.3* were prominently expressed in leaves and bracts. whereas members of the *PheSnRK3s* subfamily members were abundantly expressed in leaves, stamens, glumes, and flower buds.

**Fig. 6:**
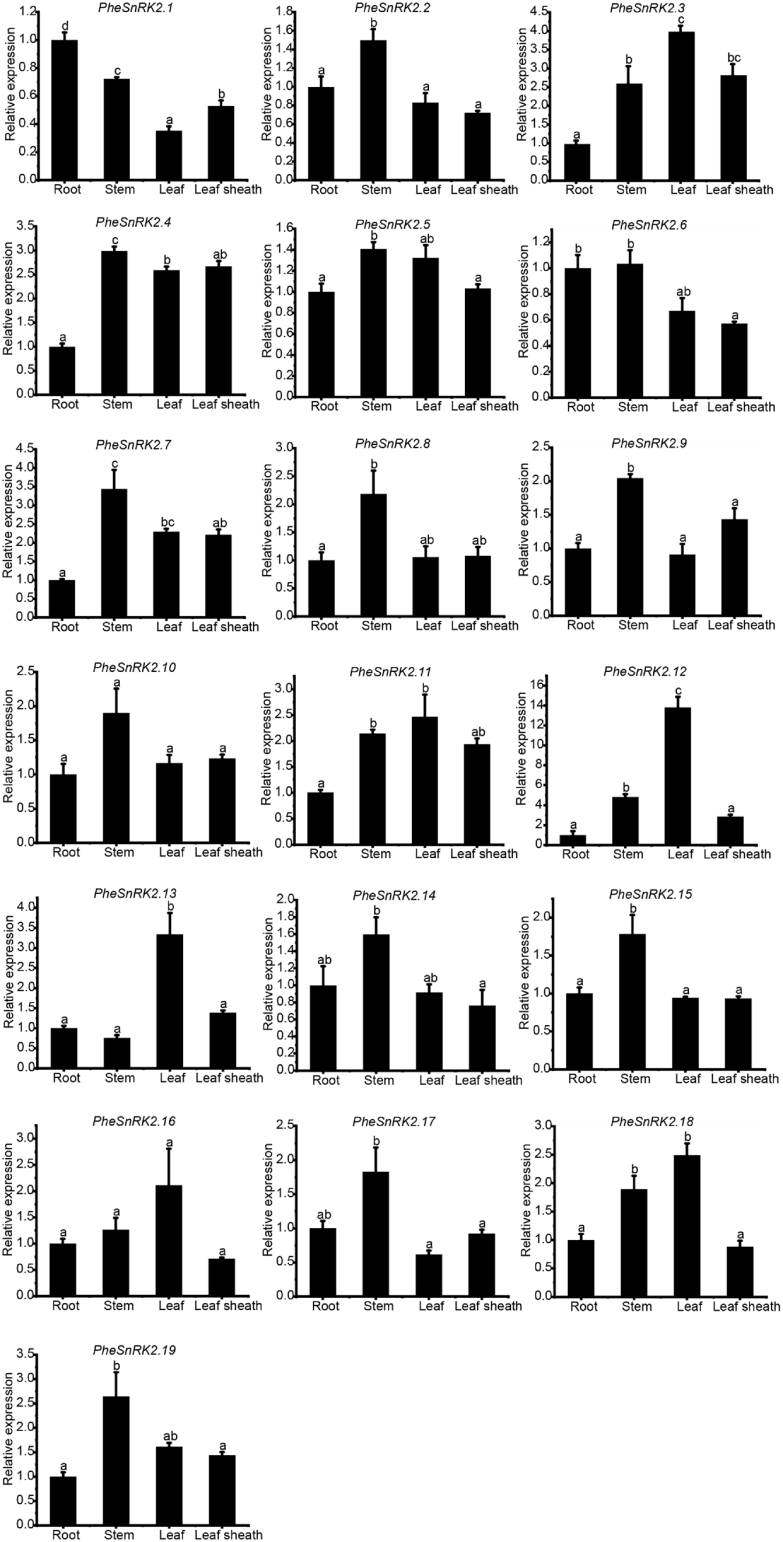
Relative expression of PheSnRK2s in different tissues of 2-month-old bamboo seedlings. The data were obtained from qRT-PCR analysis of different tissues of bamboo seedlings. Significance was evaluated using Student’s t-test, one-way ANOVA, and multiple comparisons. Column heights represent the mean relative expression levels (± SE, *n* = 3), where *p* < 0.05 indicates a significant difference. Different lowercase letters indicate significant differences

Under different sugar and hormone treatments, *PheSnRK* genes displayed differential expression patterns (Fig. 5c,d). Specific PheSnRK genes, such as *PheSnRK1.1*, *PheSnRK1.4*, *PheSnRK1.5*, *PheSnRK2.12*, and *PheSnRK3.20*, were strongly induced by sucrose yet repressed by glucose. In contrast, *PheSnRK2.1*, *PheSnRK2.16*, *PheSnRK3.8*, *PheSnRK3.23* and *PheSnRK3.35*, were exclusively upregulated in response to glucose. The treatment with the mTOR inhibitor, AZD8055, led to selective upregulation of *PheSnRK2.9* and *PheSnRK3.49*, generating overall variable gene expression responses. Four of the six genes of the *PheSnRK1s* subfamily exhibited high expression levels under IAA treatment, while shoeing low expression levels under inhibitor treatment (Fig. 5d). The majority of the genes in *PheSnRK2s* and *PheSnRK3s* subfamilies demonstrated elevated expression levels under treatment with the growth hormone inhibitor TIBA, with a few showing suppressed expression. In addition to *PheSnRK2.1* (root), *PheSnRK2.3* (leaf), *PheSnRK2.10*, *PheSnRK2.13* (leaf) and *PheSnRK2.16*, most of the *PheSnRK2s* subfamily genes were highly expressed in the stem (Fig. 6).

### Overexpression of *PheSnRK2.9* confers abiotic stress tolerance in transgenic *Arabidopsis*

**Fig. 7:**
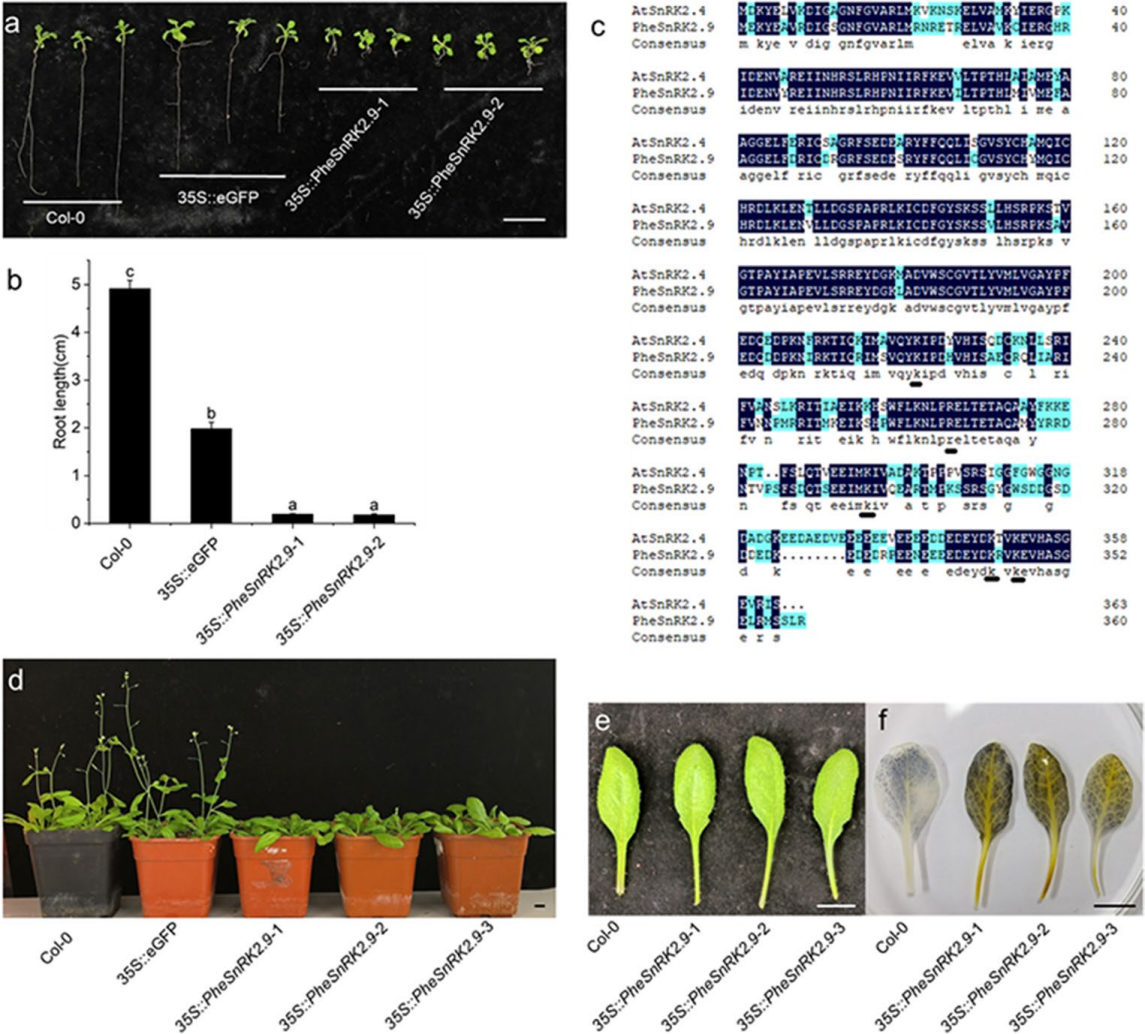
Overexpression of PheSnRK2.9 inhibited root growth and leaf starch degradation in *Arabidopsis thaliana*. **a** overexpression *PheSnRK2.9* the *Arabidopsis* root phenotype. **b** Corresponding *Arabidopsis* root length statistics in A. Significance was assessed using Student’s t-test, one-way analysis of variance, and multiple comparisons. Column height represents the average root length (± SE, *n* = 3), where *p* < 0.05 indicates significant difference. Different lowercase letters indicate a significant difference. **c** Protein sequence comparison between AtSnRK2.4 and PheSnRK2.9. DNAMAN software was used to compare the protein sequences, and the underline indicated that the candidate residues binding PA in *Arabidopsis thaliana* were also present in Moso bamboo. **d** 6-week-old *Arabidopsis thaliana* plant phenotype overexpressing *PheSnRK2.9*. **e** 6-week *Arabidopsis thaliana* rosette leaves. **f***Arabidopsis thaliana* leaf starch staining after 24 h dark treatment. Col-0: Columbia wild type; 35 S::eGFP: Transgenic line containing eGFP label; 35 S::PheSnRK2.9-1, 35 S::PheSnRK2.9-2, 35 S::PheSnRK2.9-3,: Different transgenic lines. Bars = 1 cm

We observed that root development in the overexpressed *PheSnRK2.9* line was inhibited on Kanamycin medium (Fig. 7a,b). The root system of *Arabidopsis Thaliana* was significantly shorter than that of *Arabidopsis Thaliana* with 35 S::eGFP unloaded. The PA-binding domain (PABD) present in the *Arabidopsis* AtSnRK2.4 protein was found to affect the root development of *Arabidopsis thaliana* [41].

A comparison of the PheSnRK2.9 protein sequence with the AtSnRK2.4 protein sequence revealed that lysine and arginine residues, which preferentially bind to PA, were also conserved in PheSnRK2.9 (Fig. 7c). Furthermore, compared with the control group, under dark treatment conditions, more starch was distributed in the leaves of *Arabidopsis Thaliana* overexpressing *PheSnRK2.9*, suggesting that *PheSnRK2.9* may inhibit starch degradation under starvation conditions (Fig. 7d,e,f).

**Fig. 8:**
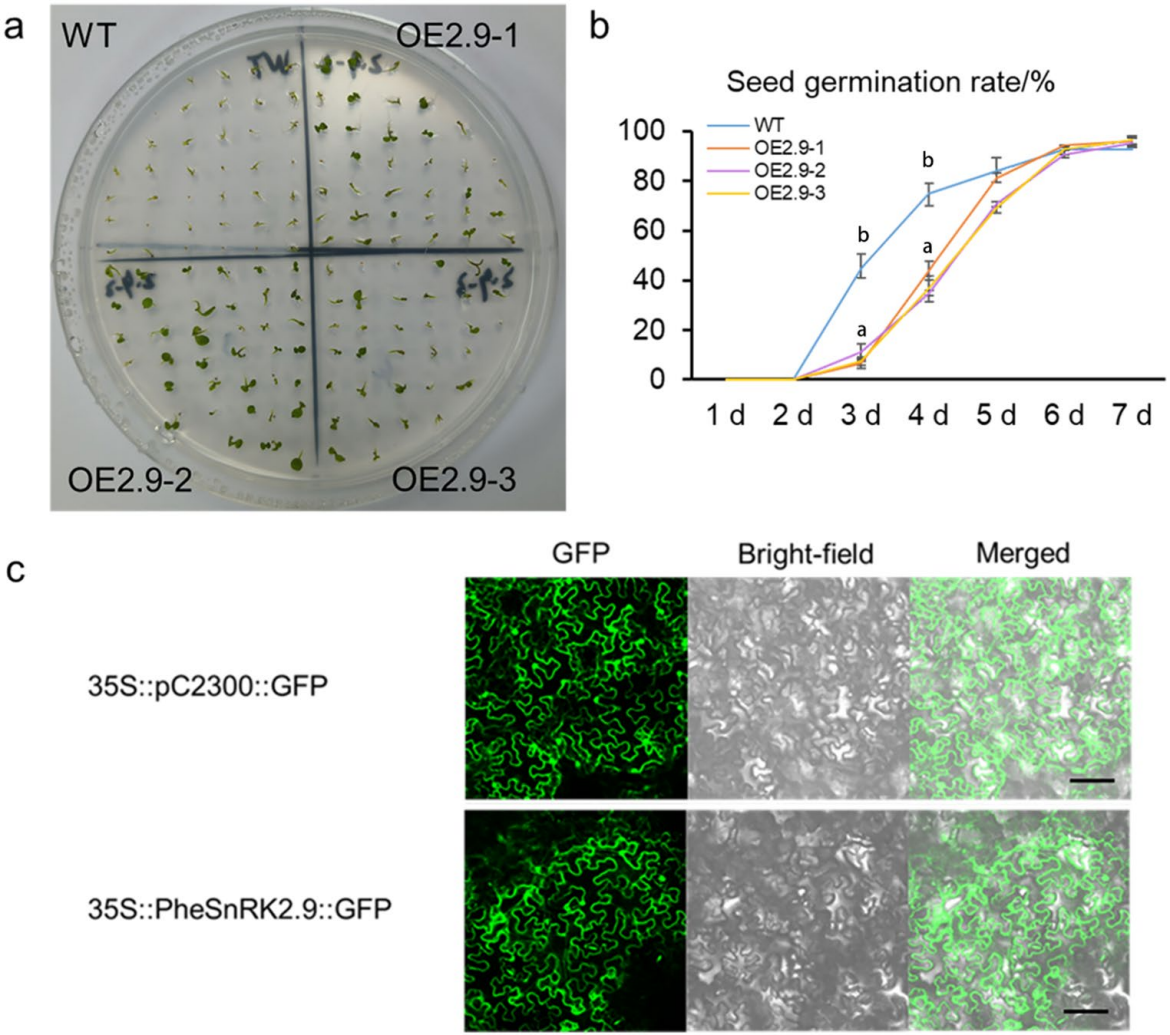
Seed germination and subcellular localization under salt stress. **a** Overexpressed *PheSnRK2.9 Arabidopsis* seed germination on a medium containing 100 mM NaCl. **b** Seed germination rate statistics. We evaluated the significance of seed germination of different transgenic lines on each day using Student’s t-test, one-way ANOVA, and multiple comparisons. (*n* = 35, ± SE), *p* < 0.05. The germination rate of the seeds on the first and second days was 0 for both, without considering inter-group differences. Starting from the third day, the inter-group differences in germination rate of transgenic seeds were found to be significant, with the following respective p-values: 0.00059 (3d), 0.00074 (4d), 0.05656 (5d), 0.51261 (6d), 0.60815 (7d). **c** Subcellular localization of PheSnRK2.9 in leaves of *Nicotiana benthamiana*. WT: Col-0; OE2.9-1/OE2.9-2/OE2.9-3: different transgenic lines. Bar = 100 μm

In order to further investigate the response of PheSnRK2.9 to salt stress, a germination experiment was conducted using overexpressed *Arabidopsis thaliana* seeds under salt stress conditions (Fig. 8a,b). The results revealed that salt stress inhibited seed germination in the early stage; however, after the fifth day, the germination rate was comparable to that of the wild type, demonstrating robust salt tolerance in subsequent growth. The results indicated that PheSnRK2.9-eGFP was localized in cytoplasm and nucleus (Fig. 8c).

**Fig. 9:**
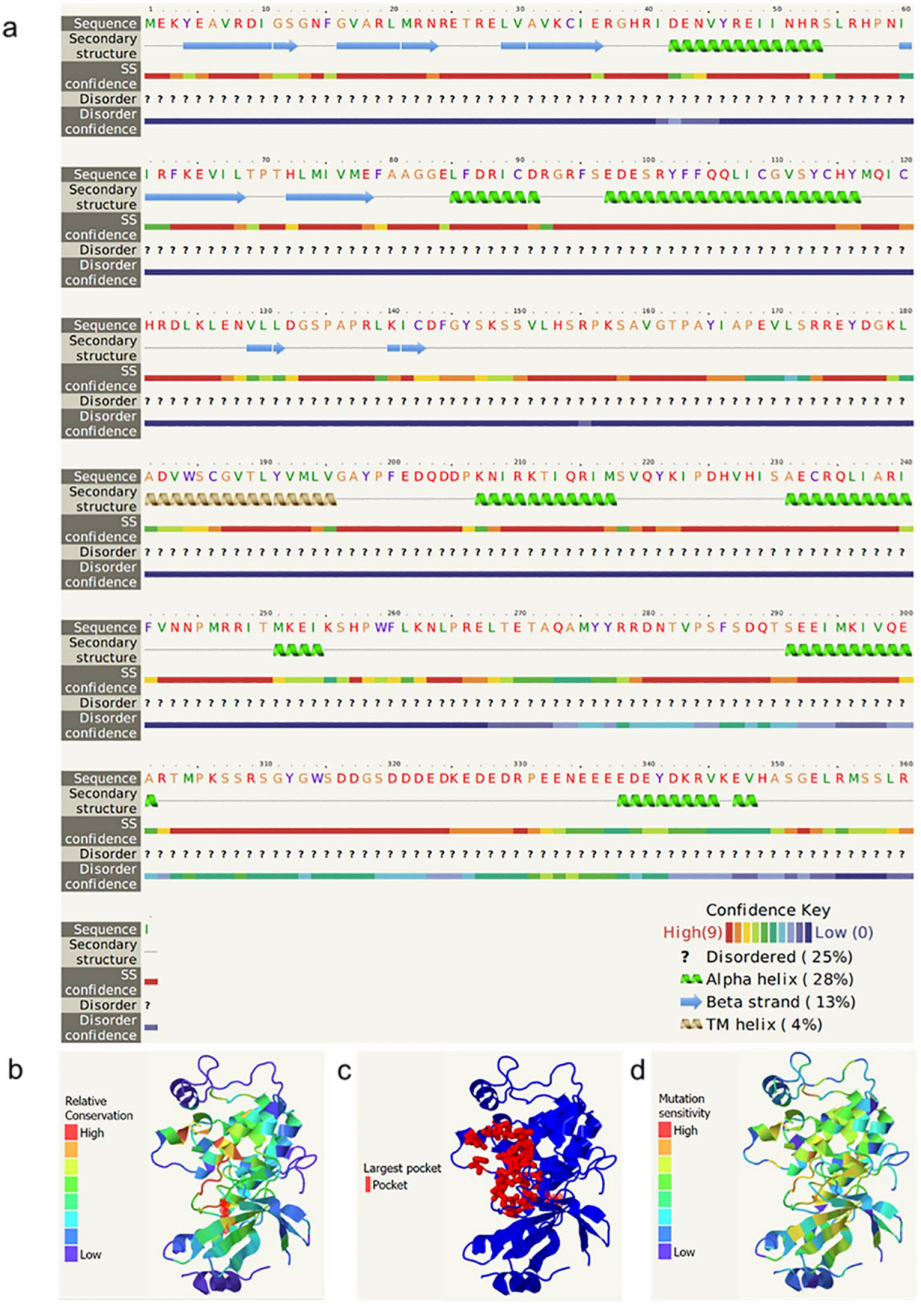
Protein structure prediction of PheSnRK2.9. **a** Secondary structure prediction of protein; **b** Amino acid conservation in the tertiary structure of protein; **c** Location of pockets in the three-dimensional structure of proteins; **d** Sensitivity to mutations in the three-dimensional structure of proteins

The predicted secondary and tertiary structures of PheSnRK2.9 are presented in Fig. 9. In the secondary structure, α-helices accounted for 28% of the sequence, and β-strands accounted for 13% (Fig. 9a). In the 3D structure, amino acids located in the pocket were found to be more conserved and sensitive to mutations (Fig. 9b,c,d), as mutations in these amino acid could significantly impact the protein’s functionality.

**Fig. 10:**
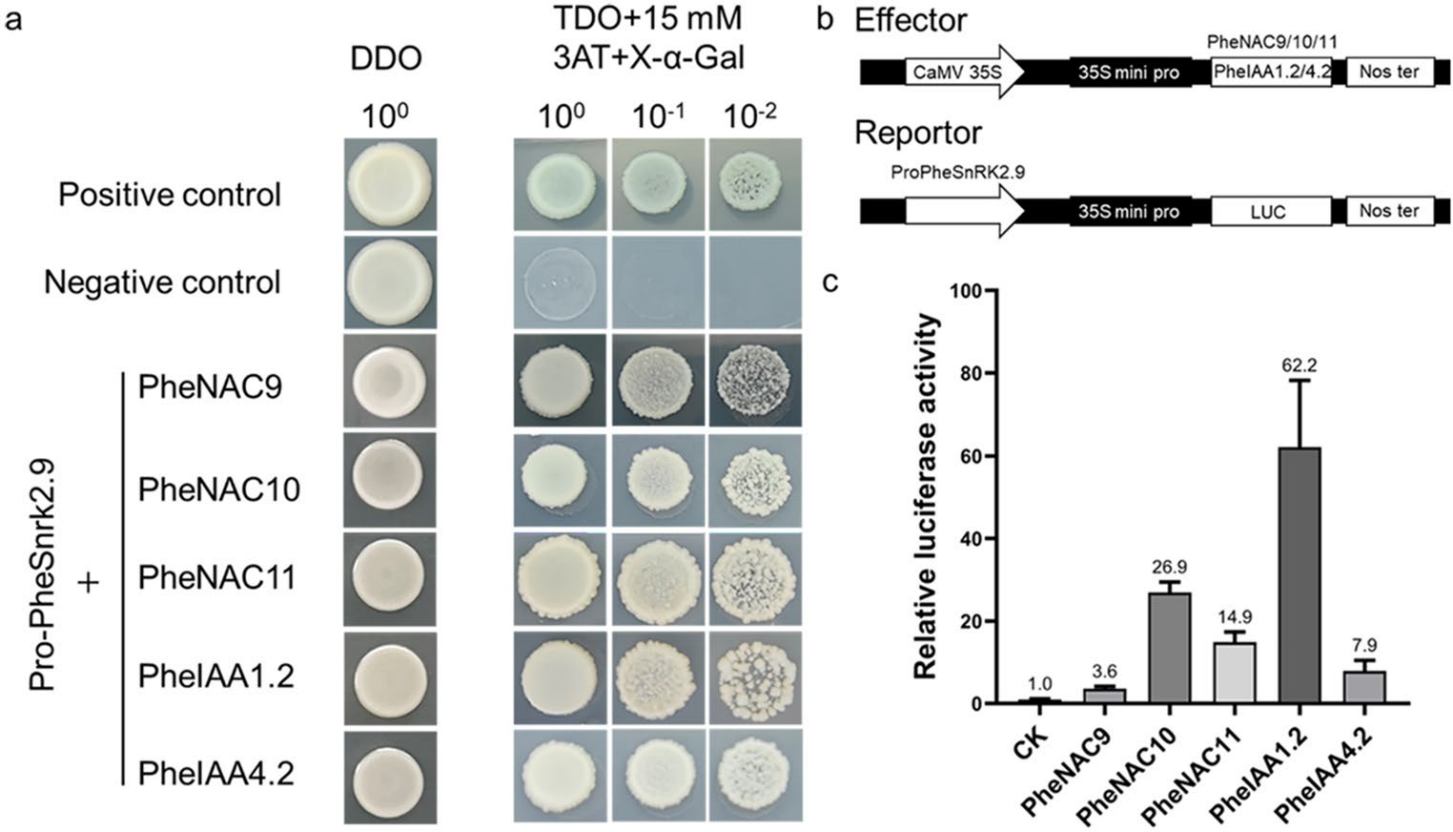
Analysis of upstream regulatory factors of *PheSnRK2.9* and verification of dual luciferase. **a** Yeast one-hybrid assay. Positive control is pGADT7 53 + pHIS2 53 and negative control is pHIS2 53 + pGADT7 Rec2. pro-PheSnRK2.9 represent the *PheSnRK2.9* gene promoter region; **b** Schematic diagram of the dual luciferase vector construction. **c** The analysis results of relative fluorescence activity. The luminescence ratio of firefly LUC to Renilla LUC was determined according to a dual luciferase reporter system. Experiments were repeated at least three times and results are expressed as mean ± standard deviation. The differences in dual luciferase enzyme activity between each transcription factor binding to the promoter and the control reached significance, with the following respective p-values: 0.00104 (PheNAC9), 0.00006 (PheNAC10), 0.00056 (PheNAC11), 0.00273 (PheIAA1.2), 0.00919 (PheIAA4.2).

To investigate the upstream regulatory factors of *PheSnRK2.9*, we screened a yeast library of Moso bamboo and identified PheNAC9, PheNAC10, PheNAC11, PheIAA1.2, and PheIAA4.2 as potential binders to the promoter of *PheSnRK2.9*. Subsequently, we confirmed through yeast one-hybrid assays that these transcription factors can indeed bind to the promoter of PheSnRK2.9 (Fig. 10a). We constructed a dual luciferase reporter system and expressed it transiently in tobacco (Fig. 10b,c). The results demonstrated that all selected transcription factors could activate the expression of PheSnRK2.9, with PheNAC10 and PheIAA1.2 exhibiting particularly robust activation, reaching 27 and 62 times the expression levels compared to the control, respectively

## Discussion

In this research, we identified and classified 75 *SnRK* family members in the Moso bamboo genome into three subfamilies, paralleling the homology classifications with *Arabidopsis* and rice. These were labeled *PheSnRK1.1* to *PheSnRK3.50* based on chromosomal positioning. Our comprehensive analysis investigated the phylogenetic relationship, gene structure, protein motifs, chromosome distribution, and promoter cis-elements of *PheSnRK* genes. We also exploited available transcriptome data to discern expression patterns of *PheSnRK* family genes and their responses to plant hormones and exogenous sugars. The study illuminated how transgenic *Arabidopsis* seeds expressing *PheSnRK2.9* exhibited enhanced salt-tolerant and were regulated by transcription factors PheNACs and PheIAAs, laying the groundwork for future investigation into the SnRK gene family’s role in plant growth and stress responses.

In the reported studies *Arabidopsis*, rice, and wheat [20, 42], 39, 50, and 174 family members were identified, respectively. In the genome of Moso bamboo, SnRK were identified a total of 75 family members. Comparative genomic analysis indicated that the number of SnRK1 subfamily members is relatively conserved across species. Moso bamboo, as a tetraploid, possesses a greater number of SnRK genes than diploid species, but fewer than hexaploid species like wheat, with the SnRK3 family exhibiting the most significant variation in gene count amongst these crops. C*is*-elements in promoters can bind to transcription factors to regulate gene expression, thus influencing plant growth and development in response to environmental changes [43]. *FvSnRK1.1* of strawberry is involved in low temperature response, light response, and fruit ripening [44]. In this study, *PheSnRKs* promoters were found to contain several types of *cis*-elements, such as auxin response, ABA response, GA response, MeJA response, SA response, as well as low temperature and light response elements. This suggests that *PheSnRKs* genes may be involved in multiple signaling response pathways to regulate the growth and development of bamboo shoots.

Considering the challenges of genetic transformation in bamboo and the fact that SnRK is an evolutionarily conserved protein kinase, we investigated gene function by transforming the model plant *Arabidopsis thaliana*. We observed that *Arabidopsis* expressing *PheSnRK2.9* showed not just salt-tolerant but also a decrease in starch degradation in leaves, highlighting the gene’s potential role in stress tolerance mechanisms.

**Fig. 11:**
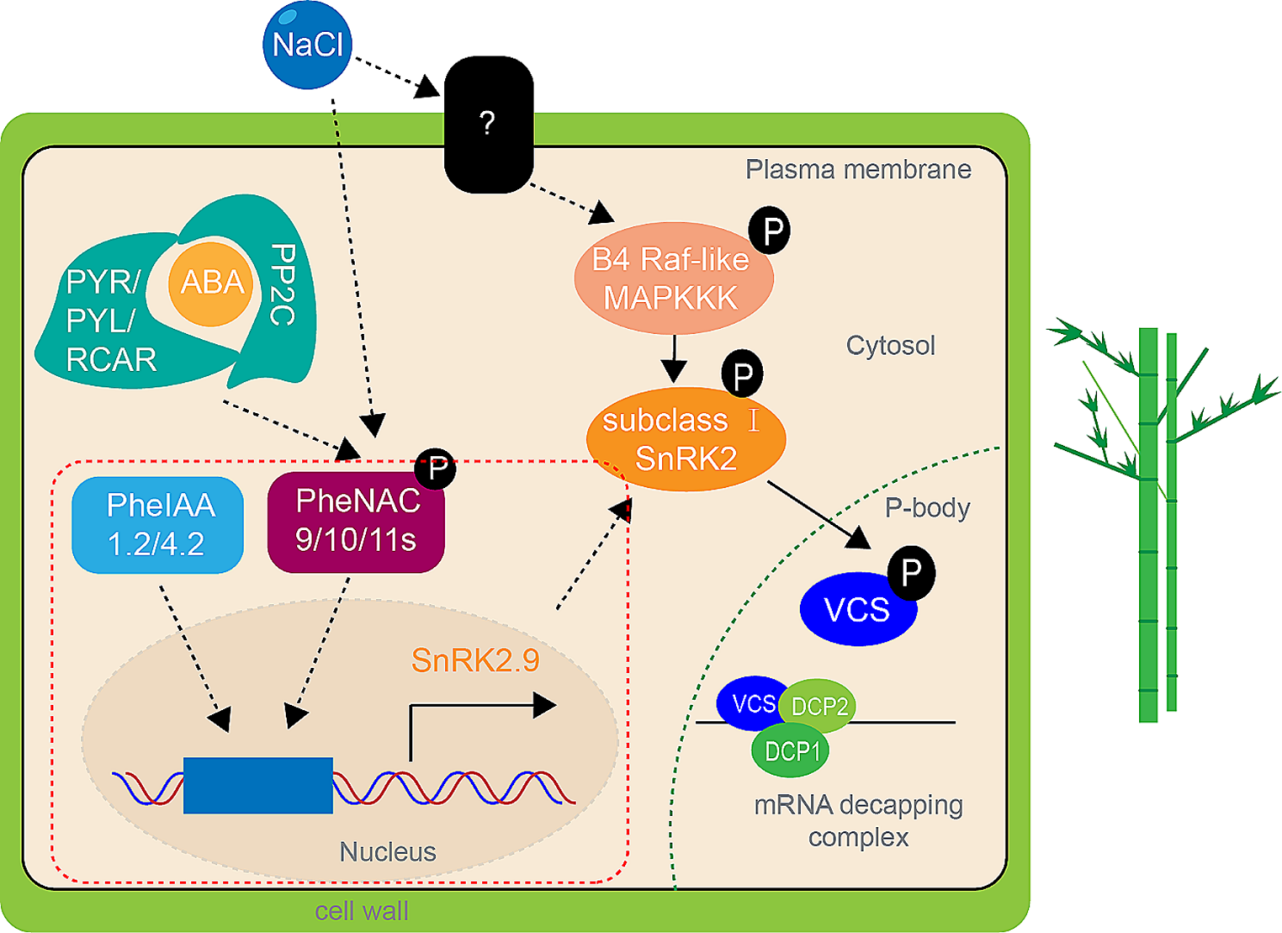
Schematic diagram of SnRK2.9 regulated by upstream transcription factors. The red dotted box is the main finding of this study

Research has reported that both salt stress and osmotic stress can activate SnRK2s protein kinases. SnRK2s transmit signals to downstream transcription factors, including ABFs, zips, MYBs, NACs, WRKYs, and AP2/ERFs, inducing the expression of stress-responsive genes [45]. Class I SnRK2s, such as SnRK2.1, SnRK2.4, SnRK2.5, SnRK2.9, and SnRK2.10, exhibit specific responses to osmotic stress independent of ABA. Currently, there is limited understanding of the upstream regulatory mechanisms of the subfamily I of SnRK2s. In Arabidopsis, B4 Raf-like MAPKKKs phosphorylate and activate subfamily I SnRK2s under osmotic stress (Fig. 11) [46]. Salt stress induces the production of phosphatidic acid (PA), which leads to the endocytosis of the auxin transporter protein PIN2. Subfamily I protein kinases of SnRK interact with PA and translocate to the processing bodies. They bind and phosphorylate VCS, an mRNA decapping protein involved in RNA stability, thereby regulating mRNA degradation [47]. Our findings contribute to a better understanding of the upstream regulatory mechanisms.

Previously, our group reported that the transcription factor PheIAA4.2, in combination with PheARF9, influences bamboo shoot development, and PheIAA16.3 interacts with PheGF14 and PheIAA27.2 [48]. The function of transcription factor PheIAA1.2 has not been further studied. After salt stress, the expressions of *PheNAC9*, *PheNAC10* and *PheNAC11* are significantly up-regulated (more than 10 times), and the up-regulation of *PheNAC10* and *PheNAC11* is more than 50 times [49]. This indicating that these three genes play an important role in the salt stress response of bamboo. Through this study, we proposed that *PheSnRK2.9* may be regulated by PheIAAs and PheNACs, which affect the growth and development and salt tolerance of bamboo (Fig. 11). The current genetic transformation system of bamboo is still immature. The transgenic experiments in this study were only conducted in *Arabidopsis*, therefore, there are still significant limitations in the genetic functional research of bamboo.

## Conclusion

In this study, we conducted a comprehensive analysis of the gene characteristics of the Moso bamboo SnRK family, thus laying the groundwork for a further understanding of the biological functions of the *PheSnRK* genes. Our results indicate that PheNAC9/10/11 transcription factors modulate the expression of *PheSnRK2.9* gene by binding to its promoter, thereby influencing both plant growth and development as well as response to abiotic stress (Fig. 11). These findings lay a theoretical groundwork for advancing our comprehension of the stress adaptation mechanism of Moso bamboo. This research provides pivotal insights into the functional complexities of the SnRK gene family and sets a promising stage for probing the molecular foundations of bamboo’s remarkable growth dynamics and resilience.

### Electronic supplementary material

Below is the link to the electronic supplementary material.

Supplementary Material 1 The primers of PheSnRK2.9

Supplementary Material 2 The characteristics of the PheSnRK gene family in *Phyllostachys edulis*

Supplementary Material 3 Cis-elements analysis PheSnRK genes of *Phyllostachys edulis*

Supplementary Material 4 Identification of overexpression of *Arabidopsisthaliana* with 35S::PheSnRK2.9

## Data Availability

Genome files of *Arabidopsis thaliana*, *Oryza sativa*, and Moso bamboo species were obtained from TAIR (https://www.arabidopsis.org/), Rice Genome Annotation Project (http://rice.uga.edu/), Bamboo (http://gigadb.org/dataset/100498) respectively. The RNA-seq data used in this study are unpublished data from the group. Someone wants to access the data of this study, please contact the corresponding author of this article.
