# Supplementary material for: Deciphering the regulatory role of PheSnRK genes in Moso bamboo: insights into hormonal, energy, and stress responses
Source: BMC Genomics. 2024 Mar 6;25:252. doi: 10.1186/s12864-024-10176-7 (PMC10916206; doi:10.1186/s12864-024-10176-7)

Figure S1. Identification of overexpression of *Arabidopsis thaliana* with 35S::PheSnRK2.9. M: 2000 bp Maker; WT: Col-0; P: Plasmid; #1 - #22 Transgenic *Arabidopsis thaliana.* The gene fusion eGFP was validated by PCR with a molecular size of 730 bp.


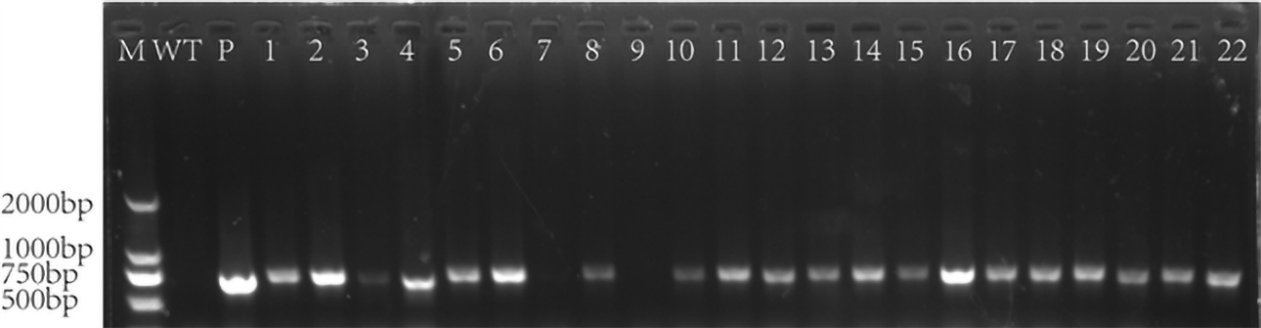

Supplement: Supplementary file 4 — Supplementary Material 4 Identification of overexpression of Arabidopsisthaliana with 35S::PheSnRK2.9 [file 12864_2024_10176_MOESM4_ESM.docx]
